# Dementia-Capable Workforce: Outcomes of a Scalable IPE Dementia Curriculum for Health Profession Students

**DOI:** 10.1093/geroni/igaf122.4094

**Published:** 2025-12-31

**Authors:** Jung Kwak, Veronica Young, Lauren El-Assad, Kwaku Duah Oppong, Sydney Silverman, Alyssa Aguirre

**Affiliations:** University of Texas-Austin, Austin, Texas, United States; The University of Texas at Austin, Austin, Texas, United States; The University of Texas at Austin, Austin, Texas, United States; The University of Texas at Austin, Austin, Texas, United States; The University of Texas at Austin, Austin, Texas, United States; The University of Texas at Austin Dell Medical School, Austin, Texas, United States

## Abstract

Health professional students require enhanced training in Alzheimer’s Disease and Related Dementias (ADRD) and interprofessional collaboration to address workforce gaps in dementia-capable care. We developed and evaluated an online interprofessional education (IPE) curriculum for students in nursing, medicine, pharmacy, social work, and speech-language pathology at a university in Texas. The curriculum included two asynchronous modules (brain health, early detection, and IPE principles) and a virtual IPE Day with case-based, team-oriented learning. Pre- and post-surveys assessed ADRD knowledge, attitudes, and IPE competencies using validated scales. Qualitative feedback was collected through open-ended responses and analyzed thematically. Of 58 students enrolled, 42 completed both modules and 30 participated in the IPE Day. Statistically significant improvements were observed in ADRD knowledge (mean increase = 1.43, p < .001) and preparedness to support families (1.49, p < .001). Significant gains were also found across five IPE competency domains: communication (mean increase = 0.48, p = .003), collaboration (0.53, p < .001), roles and responsibilities (0.43, p = .005), patient-centered care (0.50, p = .002), and team functioning and conflict management (0.39, p = .001). Over 90 percent of participants agreed the curriculum supported their learning and professional development. Qualitative analysis revealed five themes: students valued active learning strategies, clinical relevance, and self-paced flexibility, while identifying opportunities for greater interprofessional integration and extended simulation time. These findings support the feasibility and impact of brief, scalable virtual IPE curricula to enhance dementia care competencies and provide implications for interprofessional curriculum design in ADRD education and training.

